# *Ab Initio* Potentials for the Ground *S*_0_ and the First Electronically Excited Singlet *S*_1_ States of Benzene–Helium with Application to Tunneling Intermolecular Vibrational States

**DOI:** 10.1021/acs.jpca.4c01491

**Published:** 2024-07-17

**Authors:** Leonid Shirkov

**Affiliations:** Institute of Physics, Polish Academy of Sciences, Al. Lotników 32/46, 02-668 Warsaw, Poland

## Abstract

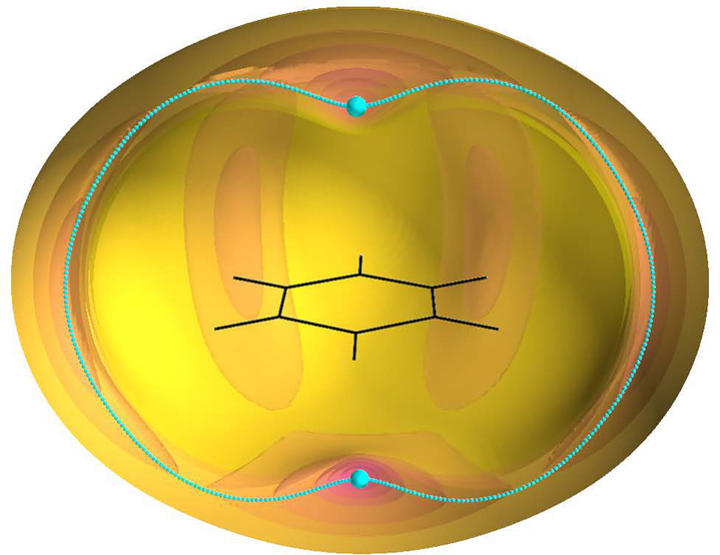

We present new *ab initio* intermolecular potential energy surfaces for the benzene–helium complex in its ground (*S*_0_) and first excited (*S*_1_) states. The coupled-cluster level of theory with single, double, and perturbative triple excitations, CCSD(T), was used to calculate the ground state potential. The excited state potential was obtained by adding the excitation energies *S*_0_ → *S*_1_ of the complex, calculated using the equation of motion approach EOM-CCSD, to the ground state potential interaction energies. Analytical potentials are constructed and applied to study the structural and vibrational dynamics of benzene–helium. The binding energies and equilibrium distances of the ground and excited states were found to be 89 cm^–1^, 3.14 Å and 77 cm^–1^, 3.20 Å, respectively. The calculated vibrational energy levels exhibit tunneling of He through the benzene plane and are in reasonable agreement with recently reported experimental values for both the ground and excited states [Hayashi, M.; Ohshima, Y. *J. Phys. Chem. Lett.***2020**, *11*, 9745]. Prospects for the theoretical study of complexes with large aromatic molecules and He are also discussed.

## I Introduction

The molecular complexes of aromatic molecules with He atoms (ArmHe) stand out from other complexes of aromatic molecules with heavier Rg atoms (ArmRg) because they are extremely weakly bound. The potential energy depth for ArmHe is about three times less than for an analogous ArmNe complex and about eight times less than for ArmAr. Thanks to low binding energies, the intermolecular bond with He is floppy and large-amplitude intermolecular vibrations with low excitation energies are observed.^1−3^ The zero-point energies of the complexes with He are lying above half of the binding energies of He; in this case, the wave functions (WF) of He motion are spread through the monomer plane, and the vibrational levels exhibit tunneling splitting. The previous experimental studies of the benzene–He (BzHe) complex,^4−9^ and other ArmHe complexes, including large PAH molecules such as tetracene, did not reveal the splitting.^10−15^ Recently, Hayashi and Ohshima reported very high-resolution UV excitation spectra of BzHe in which the splitting of the two lowest vibrational excitations for the excited state *S*_1_ was detected.^16^ Some small splitting in the rotational transitions was also noticed earlier for the pyridine–He complex,^17^ which allegedly stems from He delocalization through the monomer plane.

The ArmHe complexes have significant implications in the context of spectroscopy of aromatic molecules embedded in helium droplets. This has been extensively studied in the literature, including refs (18−22). Electronic spectroscopy of molecules in superfluid helium nanodroplets reveals interesting effects, such as vibronic bands of 0_0_^0^ zero phonon lines (ZPL) followed by broader “phonon wings” (PW) on the blue side, as described in refs (23) and (24). The existence of superfluidity in helium nanodroplets was first reported in ref (23) based on the gap between these bands. The electronic transition *S*_0_ → *S*_1_ of the aromatic molecules to the first excited single state has been observed in helium droplets for a series of aromatic molecules, as reported in refs (14, 19, and 25−31). Furthermore, a study of the impact of the rotation of a benzene molecule on the helium density distribution in a helium droplet was conducted in ref (32). In a bunch of theoretical studies, the vibrational dynamics of He in ArmHe complexes, including large PAH molecules up to pentacene, was investigated.^1−3,33−38^ In all of these studies, the potential energy surface (PES) was obtained using simple atom–atom Lennard-Jones semiempirical potentials.^39−41^ In some cases, these potentials were adjusted to reproduce experimental data, but the accuracy of the potentials should be confirmed by more reliable PESs based on *ab initio* calculations. It is possible that such potentials may differ significantly from *ab initio* potentials and inaccurately reproduce experimental results like it takes place for BzKr.^42^

The *ab initio* ground state PES for BzHe was previously reported,^5^ aiming to complement the experimental data on rotational constants and Raman scattering coefficients also presented in that study. The reported potential was then used to study the vibrational structure of the complex, and its accuracy was recently confirmed by experiment.^16^ However, our tests of the reported potential revealed some unphysical behavior in the long-range region of the potential. While this defect does not seem to affect the reported lowest vibrational energies extracted from the potential, it cannot be ignored when it comes to higher vibrational excitations and collisional dynamics.^43−45^ Therefore, a new potential for the BzHe complex in its ground state with correct behavior in the long-range region is needed. Previous theoretical studies for other ArmHe complexes where *an initio* PES was reported are rather scarce, and no vibrational structure was studied.^46,47^

Although most experimental studies of ArmRg complexes focus on the excited state *S*_1_, the ground state potential can still be useful for assigning the experimental spectrum due to the similar shapes of the potentials for both states. Nevertheless, theoretical *ab initio* potentials for the excited state *S*_1_ have been reported for a series of ArmAr complexes in previous studies,^48−51^ which unsurprisingly gave better agreement with experimental vibrational energies observed for the *S*_1_ state than their ground state counterparts. In these studies, the excited state *E*_int_ was calculated by adding the adiabatic excitation energy found with the single-reference linear-response coupled cluster (LR-CCSD) method^52,53^ to the ground state CCSD(T) potential. It is worth noting that the equation-of-motion coupled cluster (EOM-CCSD) method,^54^ which is another way of treating electronically excited states, is equivalent to LR-CCSD when it comes to calculating excitation energy values.

The objective of this work is to construct a new potential for the BzHe ground state with the correct asymptotic behavior and at a higher level of theory. Additionally, the potential for the excited state *S*_1_ will be reported. Then, both potentials will be employed to study the tunneling motion through the monomer plane using the appropriate theoretical approach. We expect that our new potentials will be useful for the community.

The structure of our article is as follows: First, the electronic structure methods used in this work are described in Section II. Next, the analytical form of the potential is presented in Section III, followed by an analysis of the vibrational structure in Section IV. Finally, future prospects and challenges in this field are discussed in Section V.

## II *Ab Initio* Methods

The intermolecular Cartesian coordinates (*x*, *y*, *z*) describing the motion of He are chosen as follows. The reference point is at the benzene center of mass, the *z*-axis coincides with the *C*_6_ symmetry axis of benzene, and the *y*-axis crosses the midpoint of two C–C bonds. The topology of potential PES is typical for BzRg complexes; it has two symmetrical global minima *M*_*z*_ above and below the benzene plane and six symmetrical local minima *M*_*y*_ in the monomer plane. The minimum-energy paths (MEPs) connect the minima through two kinds of saddle points, *S*_*x*_ and *S*_*yz*_, six of each type. These coordinates can also be transformed into the standard spherical coordinates (*r*, θ, φ).

For the calculation of the ground state *S*_0_ potential energy *E*_int_, we used the conventional CCSD(T) method with the frozen core approximation (FC) and the counterpoise correction,^55^ along with Dunning’s basis sets aug-cc-pV*X*Z (*X* = D, T, Q).^56,57^ The basis sets were augmented with 3*s*3*p*2*d*1*f*1*g* midbond functions,^58,59^ with exponents of 0.9, 0.3, and 0.1 for *s* and *p* functions, 0.6 and 0.2 for *f* functions, 0.3 for the *f* function, and 0.25 for the *g* function. The notation aug-cc-pV*X*Z+ was used for these basis sets with midbonds. The convergence of *E*_int_ was investigated near the critical points *M*_*z*_, *M*_*y*_, and *S*_*x*_ by local interpolation, and it was found that the difference between aug-cc-pVTZ+ and aug-cc-pVQZ+ in the coordinates and energy values was very small, as shown in Table 1. Therefore, the CCSD(T)/aug-cc-pVTZ+ level of theory was used for the calculation of *E*_int_ over the entire physically meaningful range of the intermolecular coordinates. It should be noted that the authors of ref (5) employed CCSD(T)/aug-cc-pVDZ+ and did not calculate some important single-point energy values in the long-range region, which led to unphysical behavior of the potential. Details of the unphysical behavior of their potential are given in the Supporting Information.

**Table 1:** Values (α, *E*_int_), Where α Is the *z*, *y*, and *x* Coordinates (in Å) of the Critical Points *M*_*z*_, *M*_*y*_, and *S*_*x*_, Respectively, and (*y*, *z*, *E*_int_) for the Saddle Point *S*_*yz*_, and *E*_int_ Is the Energy Value at These Points (in cm^–1^), Calculated Using Different Methods for the Ground *S*_0_ and the Excited *S*_1_ States of BzHe. Notations for the Basis Sets avdz=aug-cc-pVDZ and avtz=aug-cc-pVTZ Are Used.

	method	basis	*M*_*z*_	*M*_*y*_	*S*_*x*_	*S*_*yz*_
*S*_0_	CCSD	avtz+	(3.202, −74.2)	(4.739, −38.4)	(5.393, −18.5)	
	CCSD(T)	avdz+	(3.159, −90.3)	(4.729, −45.3)	(5.388, −21.7)	
		avtz+	(3.146, −89.3)	(4.685, −46.7)	(5.343, −22.4)	(2.826, 3.251, −30.4)^a^
		avqz+	(3.139, −89.9)	(4.676, −47.4)	(5.332, −22.7)	
*S*_1_	EOM-CCSD	avtz+	(3.282, −64.3)	(4.749, −41.0)	(5.452, −19.5)	
	EOM-CCSD(T)*^b^	avtz+/avdz	(3.224, −77.6)	(4.709, −48.0)	(5.375, −23.0)	(3.284, 2.957, −29.2)^a^
		avtz+/avtz	(3.223, −77.5)	(4.700, −48.8)	(5.364, −23.4)	
		avqz+/avtz	(3.216, −77.9)	(4.692, −49.5)	(5.355, −23.7)	

a Found from analytical PES; see Section III.

b Basis set used for *S*_0_ CCSD(T)/basis set used for EOM-CCSD *S*_1_; see eq 3.

The excited state *S*_1_ of the BzHe complex dissociates to monomers with the following reaction:
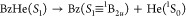
1The excited state intermolecular interaction energy *E*_int_ is calculated using the supermolecular approach:
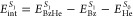
2The excited state energies *E*_BzHe_^*S*_1_^ and *E*_Bz_^*S*_1_^ can be calculated by the EOM-CCSD method. However, *E*_int_ found with CCSD for the ground state and with EOM-CCSD for the excited state is sensitive to the absence of important triple contributions. As shown in Table 1, the binding energy *D*_*e*_ for the ground state potential is severely underestimated when compared to the CCSD(T) values. On the other hand, employing the more accurate EOM-CCSD(T)^60^ or EOM-CCSDT^61^ in eq 2 would be too time-consuming in a reasonable basis set for this complex. Instead, one can use a computationally cheaper method previously applied to several ArmAr complexes for calculation of *S*_1_ potentials.^48−51^

In this approach, the adiabatic excitation energies for BzHe and Bz are found in the dimer basis as the difference between EOM-CCSD (or LR-CCSD) energies for *S*_1_ calculated using the excited state geometry of benzene and the ground state CCSD energies for *S*_0_ calculated using the ground state benzene geometry. Then, the difference between the excitation energies for BzHe and Bz is added to the ground state CCSD(T) potential, giving the excited state *E*_int_. This value has to be corrected by the difference between He energies found in the dimer basis set in the excited and ground state benzene geometries to eliminate the basis set superposition error (BSSE). It is convenient to write this approach as follows:
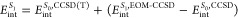
3The method described by eq 3 will be denoted as EOM-CCSD(T)* in this work. To calculate the excited state correction, we tested two basis sets, namely aug-cc-pVDZ and aug-cc-pVTZ. As shown in Table 1, the difference in energy values calculated with these two basis sets is almost negligible. Therefore, we used the smaller basis set and a *C*_1_ point group symmetry at the same He positions as for the ground state calculations. The overall number of He positions was 349 in all the calculations. The number is excessive because, after preliminary calculations, an additional set of He positions was added in the region above the zero-point vibrational energy to ensure an accurate description of the vibrational energy levels.

**Figure 1:**
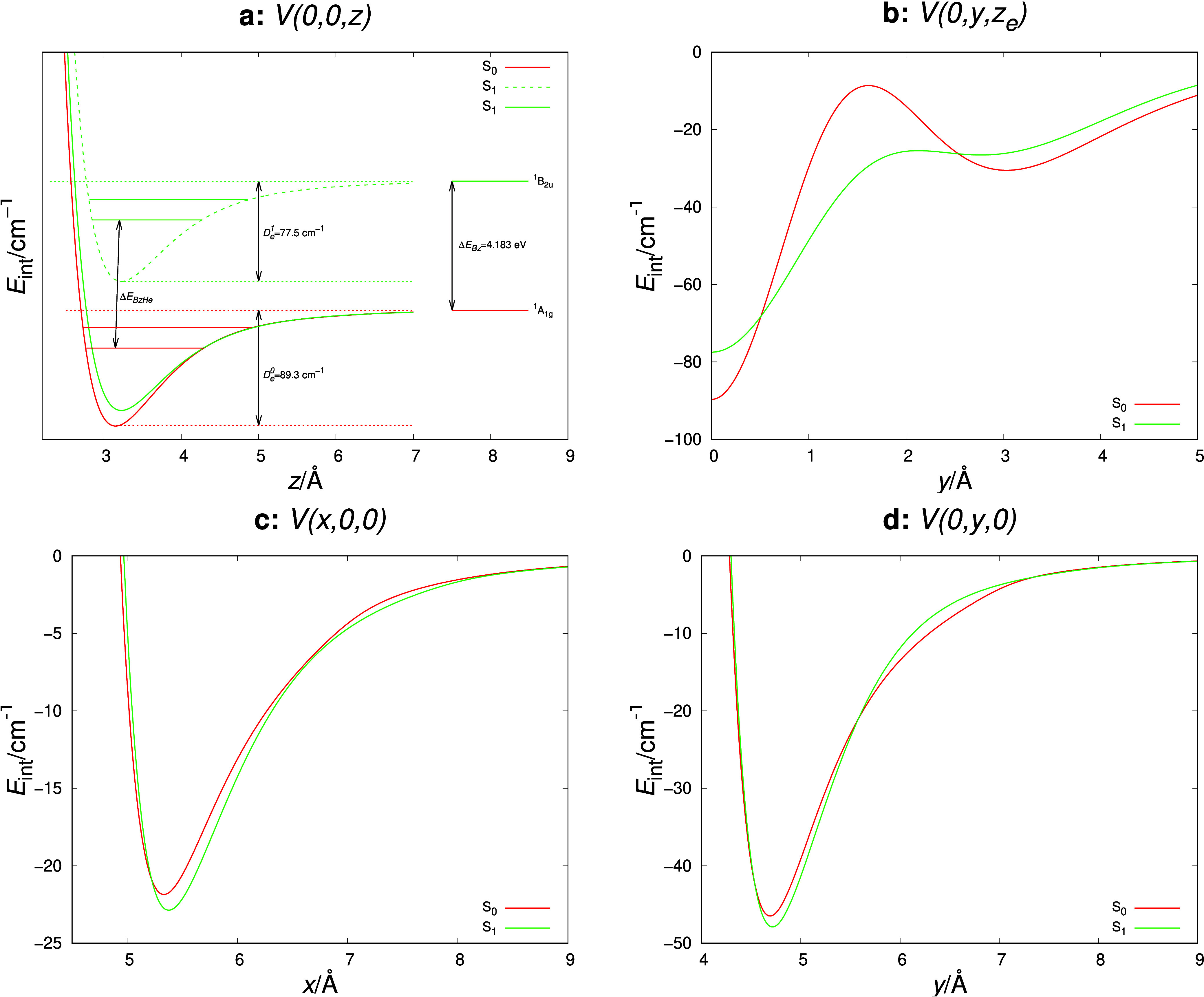
1D cuts of the ground and excited state potentials along the *x*-, *y-*, and *z*-axes and the line *x* = 0, *z* = *z*_*e*_. The solid lines denote the potential cuts normalized to zero at infinity. Δ*E*_BzHe_ and Δ*E*_Bz_ are zero–zero adiabatic excitation energies for BzHe and Bz, respectively.

The binding energy *D*_*e*_ can be found as −*V*(0,0,*z*_*e*_), where *z*_*e*_ is the equilibrium distance. This value in the excited *S*_1_ state is 12 cm^–1^ lower than its ground state counterpart, whereas the BzAr complex in the excited *S*_1_ state is characterized by a larger value of *D*_*e*_ than in the ground state.^48,49^ A comparison of the selected 1-dimensional cuts of the ground and excited state potentials is shown in Figure 1. As one can notice, the excited state potential is flatter in the vicinity of the global minimum and characterized by a lower value of the potential barrier at the saddle point *S*_*yz*_. In the monomer plane, the *E*_int_ for the ground state is slightly flatter than the excited state one.

We used the same ground state benzene geometry from ref (62) as previously for other BzRg complexes.^42,63−65^ The ground state bond lengths were 1.3915 Å for CC and 1.080 Å for CH bonds. The excited state *S*_1_ geometry was taken from ref (66) with bond lengths of 1.430 Å for CC and 1.085 Å for CH bonds. All electronic structure calculations were performed using the Molpro software package.^67^ The Supporting Information contains the calculated energy values.

## III Analytical PES

There are a few possible ways to convert the calculated set of single-point *E*_int_ to an analytical form. One way to use the expansion of *E*_int_ in spherical harmonics basis, which in the case of a nonlinear molecule and atoms has the following form:
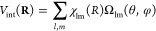
4where *m* = −*l*, −*l* + 1, ..., *l* and **R** = (*R*,θ,φ) is the vector connecting the center of mass of a nonlinear molecule and an atom. In eq 4, Ω_*lm*_(θ,φ) stands for the normalized tesseral (real) harmonics basis.^68^ In the case when the nonlinear molecule has *D*_6*h*_ symmetry, the choice of the indices (*l*, *m*) is reduced to *l* = 2, 4, 6, ... and *m* = 0, 6, 12, ... for *l* ⩾ 6.

It is convenient to divide the functions χ_*lm*_(*R*) into short- and long-range parts χ_*lm*_(*R*) = χ_*lm*_^sr^(*R*) + χ_*lm*_^lr^(*R*). The functions χ_*lm*_^sr^(*R*) and χ_*lm*_^lr^(*R*) must vanish in the long and short ranges, respectively. In this case the potential can be written as follows:
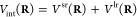
5The long-range potential can be presented using the form
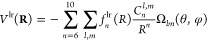
6where it is reasonable to take 1/*R*^*n*^ up to *n* = 10 for accurate reproduction of the long-range potential and the terms with (*l*, *m*) = (0, 0), (2, 0), (4, 0), (6, 0), (6, 6) due to benzene symmetry. The term with *n* = 6 describes the dispersion coupling of induced dipole polarizabilities of the monomers and the terms with *n* = 8 dipole–quadrupole and *n* = 10 quadrupole–quadrupole couplings.^65^ In order to guarantee vanishing of *V*_lr_(**R**) in the short-range regions, the expansion terms have to multiplied by damping functions *f*_*n*_(*R*). A popular choice is the Tang–Toennies functions^69^ defined as . We employ another type of damping function as explained below.

When it comes to the short-range interaction potential, we found that it is more convenient to use a many-body form of potential with variables *r*_*k*_ and functions *w*(*r*_*k*_) = 1 – e^–*a*_*k*_(*r*_*k*_–*r*_*ke*_)/*r*_*ke*_^. Here *r*_*k*_ is the distance between an atom and *k*th atom of an aromatic molecule, and *a*_*k*_ and *r*_*ke*_ are some parameters found from nonlinear optimization fitting algorithm. Such an approach was recently applied by us for a series of similar complexes.^63−65^ It can be written as follows:
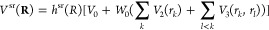
7In eq 7 the two-body terms *V*_2_ are defined as
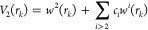
8and three-body terms as
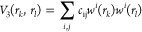
9The constants *V*_0_ and *W*_0_ are chosen manually to reproduce *ab initio E*_int_ with maximum accuracy.

For such form of the analytical potential as given in eq 7, we used another kind of damping functions for the short- and long-range parts, denoted as *h*^sr^(*R*) and *h*^lr^(*R*), used in eq 6 instead of *f*_*n*_^lr^(*R*):
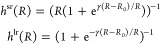
10

First, we found the set of the long-range coefficients *C*_*n*_^*l*,*m*^ by fitting *E*_int_ to eq 6 for *R* ⩾ 8 Å for both the ground and excited states PESs. *V*^lr^(**R**) reproduces the long-range *E*_int_ accurately with root-mean-square error (RMSE) of order of 0.001 cm^–1^. The obtained *C*_*n*_^*l*,*m*^ agree well with their counterparts found from the first-principles using monomer properties such as multipole moments and static/dynamics polarizabilities.^70^ More details on the comparison of *C*_*n*_^*l*,*m*^ obtained by different methods can be found in the Supporting Information.

Then, the long range part was kept frozen, and the parameters {*c*_*i*_, *c*_*ij*_, *a*_*k*_, *r*_*ke*_, *R*_0_, γ} of *V*_sr_(**R**) in eq 7 were found using the nonlinear Levenberg–Marquardt algorithm.^71,72^ The final potential described by eq 5 is characterized by RMSE equal to 0.2 (0.6) cm^–1^ in the energy range (−*D*_*e*_, 0) and 1.0 (0.6) cm^–1^ in the range (0, 500 cm^–1^) for *S*_0_ (*S*_1_) PES. Both potentials have correct asymptotic behavior in the short- and long-range regions.

**Figure 2:**
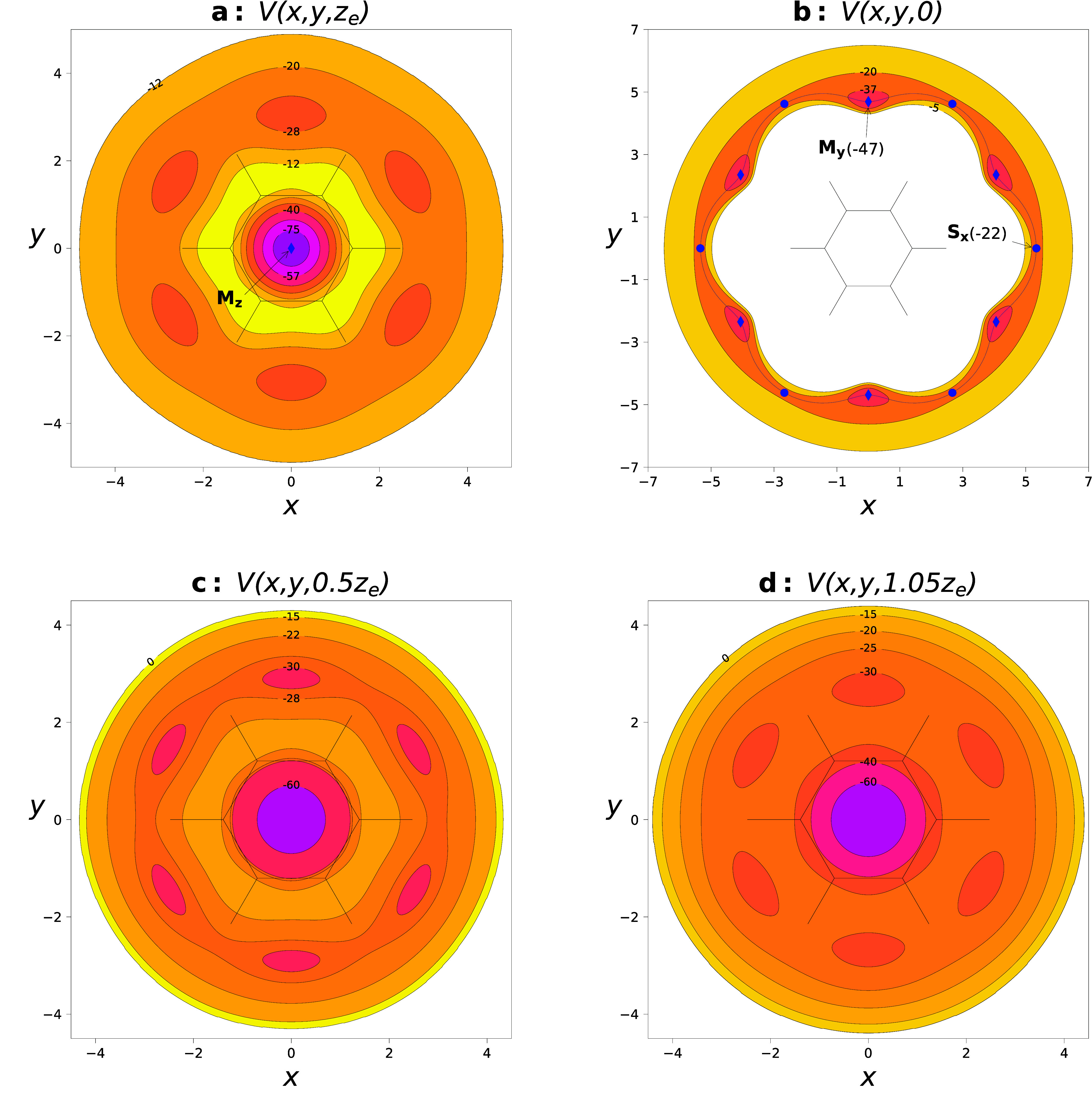
PES cuts for BzHe complex in the *xy* plane at *z* = *z*_*e*_ (a), at *z* = 0 (b), at *z* = 0.5*z*_*e*_ (c), and at *z* = 1.1*z*_*e*_ (d). The coordinates are in Å, and the contour values are in cm^–1^.

Unlike BzRg complexes with heavier Rg atoms, the BzHe potential is more anisotropic with respect to φ variable in the vicinity of the *M*_*z*_ point. The saddle points *S*_*yz*_ are located at a larger distance from the monomer plane than *M*_*z*_. The potential barrier at *S*_*yz*_ is only 58.9 cm^–1^ for the *S*_1_ PES that allows tunneling motion of He. As one can see in Figure 2a–d, the delocalization channels connect the minima *M*_*z*_ above and below the monomer plane. The saddle points *S*_*x*_ and *S*_*yz*_ are connected with the minima *M*_*z*_ and *M*_*y*_ through the minimum-energy path (MEP), shown only for the *S*_*x*_ minima in Figure 2.

A Fortran 90 program for the calculation of *E*_int_ for both PESs at a given position of He is given in the Supporting Information.

## IV Tunneling Vibrational States

**Figure 3:**
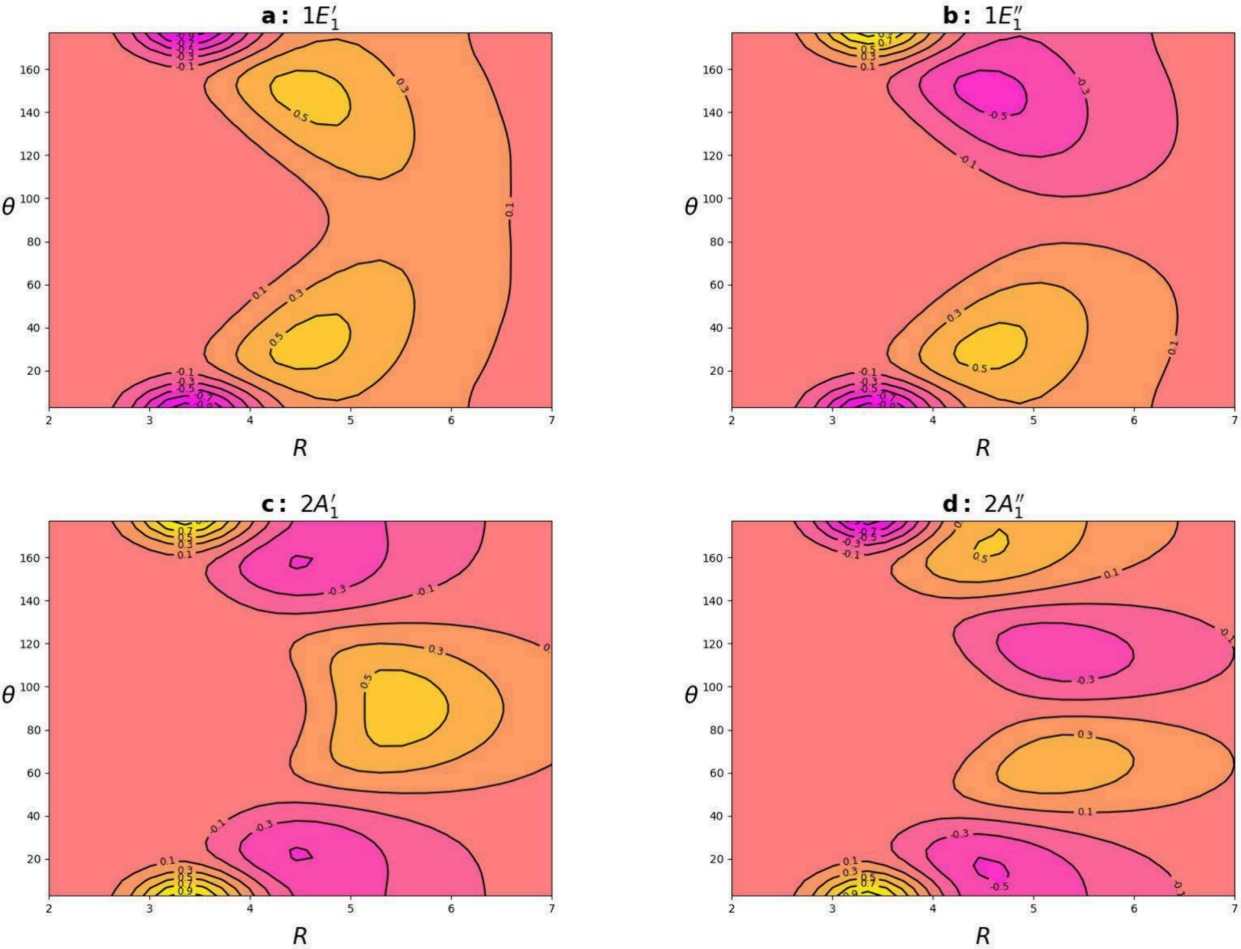
2D cuts of the normalized WFs of BzHe for the lowest vibrational states for the *S*_0_ potential at φ = 0. The WF isovalues differ by 0.2, where the maximum value of 1.0 corresponds to the WF maximum.

When a heavier rare gas atom is used in a complex with benzene, the tunneling motion of Rg can often be neglected for the lowest vibrational states, allowing for an approach using Cartesian coordinates to solve the nuclear 3D vibrational Schrödinger equation,^42,63^ in which the vibrational basis set functions are located at one global minimum *M*_*z*_. In principle, it is possible to use the approach to study the tunneling effect by locating the basis set functions either on both minima *M*_*z*_ or at the center of the coordinate frame. Instead, we employed an alternative approach that utilizes spherical coordinates (*r*, θ, φ) proposed in refs (73) and (74). Details of the numerical procedure are described in ref (63). Within the procedure, the vibrational WF are represented as a series of product basis functions constructed from stretching–bending and free internal rotation basis functions. Table 2 contains the lowest vibrational energy levels for BzHe in both considered electronic states. The results in Table 2 are given with labels for the symmetry group *G*_24_ used in ref (5) for compatibility of the results. The zero-point energies are given relative to dissociation and are found as the difference between the *D*_*e*_ values and the ground intermolecular vibrational state. Figure 3 contains cuts of the vibrational wave functions for the selected lowest states at φ = 0. The redistributions below and above the monomer plane clearly indicate the presence of quantum tunneling effect.

**Table 2:** Vibrational Energy Values (in cm^–1^) of BzHe for the Ground and Excited States

		S_0_			S_1_	
*n*	Γ_6*h*_	Lee et al.^5^	this work	expt^16^	this work	expt^16^
0	*A*_1_^′^/*A*_1_^″^	59.61	59.47		46.12	
1	1*E*_1_^′^/1*E*_1_^″^	15.75/15.97	15.28/15.55	16.0	14.757/14.761	12.9
2	2*A*_1_^′^/2*A*_1_^″^	16.26/16.90	15.55/16.33		17.91/18.56	15.88/16.48
3	1*E*_2_^′^/1*E*_2_^″^	17.45/19.72	16.80/19.10		18.76/22.37	
4	2*E*_1_^′^/2*E*_1_^″^	17.47/19.90	17.01/19.40		18.43/22.28	
5	1*B*_2_^′^/1*B*_2_^″^	17.51/20.89	17.23/20.42		19.08/23.53	
6	3*A*_1_^′^/3*A*_1_^″^	18.67/21.00	18.03/20.50		19.14/23.00	

In contrast to BzRg with heavier than He atoms,^64,65^ the assignment of the vibrational states in terms of the harmonic oscillator quantum numbers (*n*_*s*_, *n*_*b*_, *l*) for the singly degenerate stretching mode *n*_*s*_ and the doubly degenerate bending mode (*n*_*b*_, *l*) can only be done formally. The states 2*A*_1_^′^/2*A*_1_^″^ and 1*E*_1_^′^/1*E*_1_^″^ in Table 2 are approximately identified as the lowest stretching and bending modes, respectively.

The results for the *S*_0_ PES are very close to those obtained by Lee et al., though not entirely identical. Several reasons account for this disparity. First, we utilized a larger basis set for our CCSD(T) calculations, encompassing a wider set of He positions. Second, our potential exhibits correct asymptotic behavior, which may affect the higher vibrational states close to the dissociation limit. Third, the theoretical approach to treat the vibrational problem differs from that employed by Lee et al.

Our results for the *S*_1_ PES revealed the following: the value of the lowest vibrational state, corresponding to the nominal bending mode, is almost 2 cm^–1^ closer to the experimental value than its *S*_0_ counterpart. The splitting value of the vibrational energy levels extracted from the *S*_1_ PES is in good agreement with the experimental estimation of <0.02. However, the subsequent state, corresponding to nominal stretching for the *S*_1_ state, exhibit less favorable agreement with the experimental values of the vibrational energy.

We have also calculated the values of averaged intermolecular distances *R*_0_ for the zero-point vibrational energies, which are 3.590 and 3.604 Å in the *S*_0_ and *S*_1_ states, respectively. These values agree reasonably well with their counterparts derived from the experiment: 3.602 and 3.665 Å, respectively.^6^

The vibrational energy levels for the C_6_D_6_ isotopomer for both electronic states, as well as the energy levels for higher vibrational energies than those given in Table 2, are reported in the Supporting Information.

## V Conclusions

We have presented a study of the BzHe complex in its ground *S*_0_ and the first singlet excited electronic states *S*_1_. The conventional CCSD(T) method with augmented Dunning’s triple-ζ basis set supplemented by midbond functions has been employed to calculate the ground state *E*_int_. We obtain the excited state *E*_int_ by adding the adiabatic correction, found using the EOM-CCSD method, to the ground state CCSD(T) potential. A nonlinear optimization algorithm is used to construct analytical potentials for each state. Our potential for the BzHe ground state has the correct behavior in the long-range region compared to the previously reported potential.^5^

Both potentials exhibit similar shapes, although the excited state potential appears visibly flatter in the vicinity of the global minimum. We apply these potentials to solve the 3D vibrational Schrödinger equation, which describes the motion of an atom with respect to a plane symmetric top molecule using spherical coordinates. This approach enables us to study the tunneling motion of an atom through the monomer plane. The calculated vibrational energy levels exhibit splitting, and the vibrational wave functions are dispersed throughout the monomer plane. The vibrational energy values obtained from these potentials reasonably agree with recently observed experimental values^16^ for both electronic states, *S*_0_ and *S*_1_. However, the somewhat surprising disagreement in the vibrational energy value corresponding to nominal stretching for the *S*_1_ state necessitates further investigation. This discrepancy may stem from inaccuracies in the single-reference excited states methods, such as EOM-CCSD, particularly in regions closer to the dissociation limit, and multireference methods may help to remove this inaccuracy.^75^

A problem of great importance is the calculation of PES for complexes containing large aromatic molecules such as tetracene and coronene with He.^22^ Unfortunately, the “gold-standard” CCSD(T) becomes too time-consuming for such complexes. Therefore, there is a need for cheaper methods that provide similar accuracy at much less computational cost. We recently showed that symmetry-adapted perturbation theory based on density functional description of monomer properties (DFT-SAPT) can be reduced to an approximate model^76^ in which the time-consuming dispersion term can be replaced by its empirical counterpart.^77^ Another alternative is the second-order Møller–Plesset perturbation theory which, according to our preliminary tests, gives good agreement of *E*_int_ with CCSD(T) for ArmHe and ArmNe complexes thanks to fortuitous error cancellation. A detailed study of the potentials for complexes with larger aromatic molecules will be presented in a separate study.

## Supporting Information Available

Figure S1 and Tables S1–S2 (PDF)
calculated and fitted *E*_int_ for *S*_0_ and *S*_1_ states; Fortran 90 program for the calculation of *E*_int_ for both potentials at a given position of He (TXT)
