# Supplementary material for: Ab Initio Potentials for the Ground S0 and the First Electronically Excited Singlet S1 States of Benzene–Helium with Application to Tunneling Intermolecular Vibrational States
Source: J Phys Chem A. 2024 Jul 17;128(30):6132–9. doi: 10.1021/acs.jpca.4c01491 (PMC11299187; doi:10.1021/acs.jpca.4c01491)
Supplement: Supplementary file 1 — jp4c01491_si_001.pdf [file jp4c01491_si_001.pdf]

Supplementary Material to "*Ab initio* Potentials for the Ground S_0 and the First Electronically Excited Singlet S_1 States of Benzene-Helium with Application to Tunneling Intermolecular Vibrational States"

Leonid Shirkov*

Institute of Physics, Polish Academy of Sciences, Al. Lotników 32/46, 02-668 Warsaw, Poland

I. Comparison of our potential with those previously reported for some regions of the PES

Fig. S1 contains the comparison of the potential reported in Ref. [1] with ours. As one can see, the earlier potential exhibits unphysical behavior in the benzene (x, y) plane. The potential reported in our work has correct behavior in the whole physically meaningful range.

FIG. S1: Comparison of the potential by Lee *et al.* to the potential reported in this work for BzHe ground state

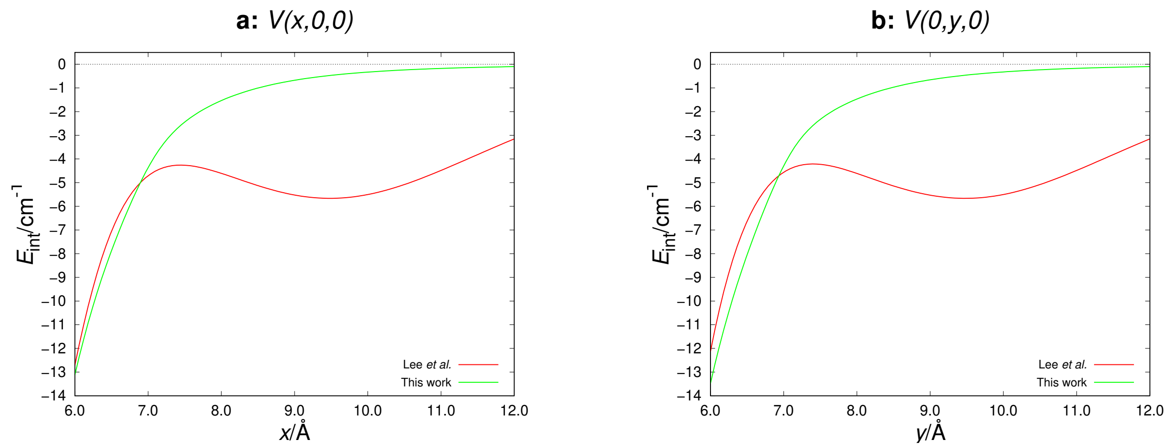

II. Long-range coefficients

The long-range induction and dispersion coefficients $C_n^{l,m} = C_{n,\mathrm{disp}}^{l,m} + C_{n,\mathrm{ind}}^{l,m}$ from eq. (6) in manuscript were calculated using the first principles using benzene multipole moments Q_m^l and static/dynamic polarizabilities $\alpha_{mm'}^{ll'}(i\omega)$ for benzene and He. More details are given in Ref. [2]. Table S1 contain comparison of the first principles coefficients with those found from fitting E_{int} calculated with CCSD(T) and EOM-CCSD(T)*.

TABLE. S1: The leading long-range coefficients $C_n^{l,m}$ (a.u.) found from fitting CCSD(T) and EOM-CCSD(T)* E_{int}, and from the first principles using DFT response and XCCSD for the BzHe potential

	S_1		S_0	
	EOM-CCSD(T)*	CCSD(T)	XCCSD	DFT resp.
$C_6^{0,0}(\times 10^1)$	4.820	4.753	4.984	4.890
$C_6^{2,0}(\times 10^1)$	-0.740	-0.909	-1.496	-1.453
$C_8^{0,0}(\times 10^3)$	3.811	3.015	3.146	3.190
$C_8^{2,0}(\times 10^3)$	-10.779	-9.365	-8.093	-8.212

* leonid.shirkov@ifpan.edu.pl

III. Vibrational energy values for isotopomers of the BzHe complex

Below are the vibrational energy levels up to dissociation limits for S_0 and S_1 for C_6H_6 and C_6D_6 isotopomers.

TABLE. S2: Vibrational energy values (in cm^{-1}) for isotopomers of BzHe in the ground S_0 and excited states S_1

		S_0		S_1	
n	Γ_{6h}	C_6H_6	C_6D_6	C_6H_6	C_6D_6
0	A_1'/A_1''	59.47	58.71	46.12	46.52
1	$1E_1'/1E_1''$	15.28/15.55	15.62/15.75	14.757/14.761	14.547/14.549
2	$2A_1'/2A_1''$	15.55/16.33	16.16/16.81	17.91/18.56	18.30/18.73
3	$1E_2'/1E_2''$	16.80/19.07	17.38/19.41	18.76/22.37	19.19/22.46
4	$2E_1'/2E_1''$	17.01/19.41	17.35/19.53	18.43/22.28	18.83/22.47
5	$1B_2'/1B_2''$	17.23/20.43	17.81/20.64	19.08/23.53	19.50/23.88
6	$3A_1'/3A_1''$	18.03/20.49	18.34/20.62	19.14/23.00	19.27/23.12
7	$2E_2'/2E_2''$	21.37/23.00	21.54/23.31	23.34/24.44	23.22/24.65
7	$3E_2'/3E_2''$	24.18/25.97	24.05/25.93	25.57/28.72	25.24/28.64
8	$2B_2'/2B_2''$	21.59/23.49	22.06/23.67	24.00/27.05	24.07/27.07
9	$3E_1'/3E_1''$	22.47/24.91	22.38/25.09	24.35/26.22	24.30/26.28
10	$4A_1'/4A_1''$	23.63/27.10	23.55/26.81	25.97/28.25	25.87/28.35
11	$4E_1'/4E_1''$	26.08/28.57	25.71/28.42	27.19/30.08	26.85/30.30
12	$5A_1'/5A_1''$	27.12/27.67	27.39/27.57	28.82/28.89	28.58/28.60

[1] S. Lee, J. S. Chung, P. M. Felker, J. L. Cacheiro, B. Fernández, T. B. Pedersen, and H. Koch, J. Chem. Phys. **119**, 12956 (2003).
[2] L. Shirkov and M. Tomza, J. Chem. Phys. **158**, 094109 (2023).
